# Life cycle assessment and energy comparison of aseptic ohmic heating and appertization of chopped tomatoes with juice

**DOI:** 10.1038/s41598-021-92211-1

**Published:** 2021-06-22

**Authors:** Sami Ghnimi, Amin Nikkhah, Jo Dewulf, Sam Van Haute

**Affiliations:** 1 Université Claude Bernard Lyon 1, CNRS, LAGEPP UMR 5007, 43 Bd 11 Novembre 1918, 69622 Villeurbanne, France; 2 ISARA Lyon, 23 rue Jean Baldissini, 69007 Lyon, France; 3 Department of Food Technology, Safety and Health, Ghent University, Faculty of Bioscience Engineering, Ghent, Belgium; 4 Department of Environmental Technology, Food Technology and Molecular Biotechnology, Ghent University Global Campus, Incheon, South Korea; 5 Department of Green Chemistry and Technology, Ghent University, Coupure Links 653, 9000 Ghent, Belgium

**Keywords:** Climate change, Environmental sciences

## Abstract

The energy balance and life cycle assessment (LCA) of ohmic heating and appertization systems for processing of chopped tomatoes with juice (CTwJ) were evaluated. The data included in the study, such as processing conditions, energy consumption, and water use, were experimentally collected. The functional unit was considered to be 1 kg of packaged CTwJ. Six LCA impact assessment methodologies were evaluated for uncertainty analysis of selection of the impact assessment methodology. The energy requirement evaluation showed the highest energy consumption for appertization (156 kWh/t of product). The energy saving of the ohmic heating line compared to the appertization line is 102 kWh/t of the product (or 65% energy saving). The energy efficiencies of the appertization and ohmic heating lines are 25% and 77%, respectively. Regarding the environmental impact, CTwJ processing and packaging by appertization were higher than those of ohmic heating systems. In other words, CTwJ production by the ohmic heating system was more environmentally efficient. The tin production phase was the environmental hotspot in packaged CTwJ production by the appertization system; however, the agricultural phase of production was the hotspot in ohmic heating processing. The uncertainty analysis results indicated that the global warming potential for appertization of 1 kg of packaged CTwJ ranges from 4.13 to 4.44 kg CO_2_eq. In addition, the global warming potential of the ohmic heating system ranges from 2.50 to 2.54 kg CO_2_eq. This study highlights that ohmic heating presents a great alternative to conventional sterilization methods due to its low environmental impact and high energy efficiency.

## Introduction

Life Cycle Assessment (LCA) is a recognized standardized methodology for examining environmental consequences in food systems. In recent years, this method has been applied to the environmental assessment of agricultural systems, such as apple^1^, peanut^2^, strawberry^3^, cacao^4^, kiwifruit^5^, canola^6^, tea^7^, peach^8^, apricot^9^, barley^10^, corn^11^, and tobacco^12^. However, there are few studies on the LCA of food processing systems, such as tomato-based product processing^13^, olive oil^14^, tea processing^15^, whole peeled canned tomato^16^, pasta production^17^, legume processing and packaging^18^, and apple juice^19^. Life cycle environmental impact assessment of food production (agricultural phase and processing) throughout its supply chain can improve the understanding of environmental impacts and determine the environmental hotspots of production systems.

In recent years, the development of an environmentally sustainable food supply chain has become important^20^. In this regard, LCA can also help policymakers and managers produce their products in a more environmentally friendly manner. In this regard, tomato (*Lycopersicon esculentum*) is the second most important vegetable crop after potato, and the total worldwide tomato production was 182 million tons in 2017^21^. There are different tomato-based products, such as purée, paste, juice, chopped tomatoes, and peeled tomatoes in tomato juice, resulting in various processing systems.

The environmental impacts of the agricultural phase of tomato production have been widely investigated in different parts of the world, such as Australia^22^, Albania^23^, Colombia^24^, Iran^25,26^, Italy^16,27^, Spain^28–30^, and Canada^31^. More information regarding the LCA studies on the agricultural phase of tomato production systems can be found in Pineda et al.^32^. However, there are a few published documents related to the LCA of different tomato processing systems. Table 1 displays a summary of the relevant literature on the LCA of tomato processing systems.

**Table 1:** Summary of the literature on the LCA of tomato processing.

Product	The studied region	Functional unit	Impact assessment methodology	Focus of the research	Environmental hotspots	References
Paste and diced tomatoes	United States	One kilogram of canned, consumer-ready tomato paste	Not available	The environmental consequences of regional and national-scale food systems	Processing and retail packaging	Brodt et al. (2013)^58^
Chopped tomatoes and peeled tomatoes in tomato juice and tomato purée	Italy	One kg of packaged product	CML 2001	Environmental impacts of various tomato based products	Agricultural phase and packaging	Del Borghi et al. (2014)^13^
Packaged tomato puree	Northern Italy	700 g puree jar	CED, CML 2001 and ReCiPe	Environmental impacts of various phases of packaged tomato puree production process	Packaging and agricultural phases	Manfredi and Vignali (2014)^49^
Tomato puree	Northern Italy	One kg of tomato purée	ILCD method	Anaerobic digestion of by-products	Packaging and agricultural phases	Bacenetti et al. (2015)^55^
Tomato juice	German Institute of Food Technologies, Germany	One kg of packaged tomato juice	Not available	Thermal, high pressure processing, and pulsed electric fields technologies compression	Packaging	Aganovic et al. (2017)^59^
Packaged peeled tomatoes	Italy	1 kg of processed tomato	ReCiPe	Pulsed electric fields technology at an industrial scale	Canning	Arnal et al. (2018)^50^
Fresh and dried organic tomato	Southern Sweden	One tonne	ReCiPe midpoint	Environmental impacts of fresh and dried tomato supply chain	Agricultural phase, packaging and drying	Bosona, and Gebresenbet, (2018)^60^
Tomato puree	Iran	500 g packaged tomato puree in a steel can with a plastic cap	CML-IA baseline	Comparison of different tomato puree production phases	Packaging	Shahvarooghi Farahani et al. (2019)^51^
Tomato-pasta sauce	The USA	1 kg product eaten at the consumer level	ReCiPe 2016	Cradle to grave environmental impact assessment	Processing and the agriculture phase	Parajuli et al. (2020)^48^
Tomato ketchup	Austria	3.8 kg ketchup	Combination of a few methodologies	Packaging systems in the light of food waste	Packaging	Wohner et al. (2020)^61^
Chopped tomatoes with juice	Italy	One kg of packaged CTwJ	CML-IA baseline, ILCD 2011 Midpoint, EDIP 2003, EDP 2013, ReCiPe midpoint, and IMPACT 2002+	Processing upon appertization and ohmic heating systems	–	Current Study

Industrial food processing is the second most notable phase of the food supply chain, accounting for 28% of the total energy use^33^. Moreover, industrial production, together with logistics and packaging (beyond the farm gate), are responsible for approximately half of the total energy consumption in the food chain^33^. The EU food industry is making significant contributions to improve energy efficiency while optimizing production processes through different approaches: (i) energy and heat recovery, (ii) selection of renewable energy sources to minimize the impact of energy consumed, and (iii) development and application of new sustainable “green and innovative” techniques in food processing. In comparison to conventional food processing technologies, green and innovative food processing technologies involve less processing time, reduced solvent and energy consumption, and a lower CO_2_ footprint^34^. The most common innovative thermal and non-thermal technologies in the food industry are ohmic heating, microwave heating, radiofrequency, high-pressure processing, and pulsed electric fields. Ohmic heating provides a rapid and uniform heating, consequentially reducing thermal damage compared to conventional heating and allows manufacturers to obtain high-quality products with minimum nutritional, sensorial, and structural changes^35^. The conversion of electric energy into thermal energy during ohmic heating results in high energy efficiency (i.e., > 90%), which is considerably higher than those achieved by the traditional indirect heating technologies that rely on the burning of fuels, such as appertization, tubular heat exchangers, and plate heat exchangers^36,37^. In a study, Aganovic et al.^38^ investigated the energy consumption and environmental life cycle of thermal, high-pressure processing (HPP), and pulsed electric field (PEF) technologies for tomato and watermelon preservation. The results indicated that the tomato juice farm-to-gate environmental impact was higher than that of watermelon juice, and the largest energy uptake was documented for HPP, followed by PEF and traditional thermal processing.

Italy leads tomato production in the EU with a share of 36%; the amount of total tomato production was 5.6 million tons (96% for processing) in 2015^39^. Reviewing the related relevant literature, it has been highlighted that in-depth research has not yet been conducted on the environmental impacts of chopped tomatoes with juice (CTwJ) processing by conventional processing, including appertization and innovative electro-technology such as ohmic heating. Similarly, for energy efficiency, research studies focus only on specific unit operations and not on the entire processing line to determine energy consumption. Thus, this study aimed to perform an energy efficiency comparison (global line and thermal unit operations) and LCA of ohmic heating and appertization for CTwJ processing.

## Materials and methods

### Tomato processing

**Figure 1:**
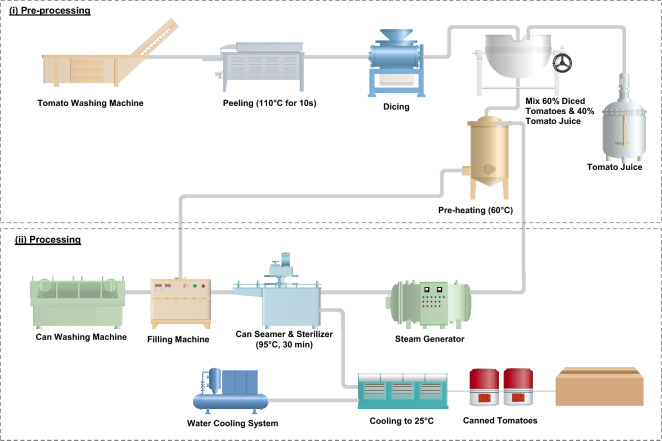
Appertization line with 6 t/h capacity, including pre-processing, canning, sterilization and cooling.

**Figure 2:**
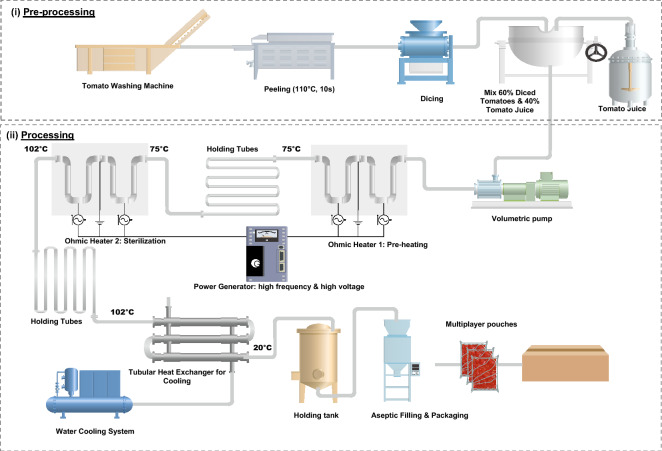
Aseptic Ohmic heating line with 4 t/h capacity, including pre-processing, preheating, sterilization, cooling and aseptic packaging.

Tomatoes (*Solanum lycopersicum *var.* Hybrid*) were grown in the Italian Puglia region. They were purchased and transported to processing plants located 160 km away and then processed at a local Italian factory. For the purpose of this study, the industrial appertization and aseptic ohmic heating units were selected as described in Figs. 1 and 2, respectively. The preparation processes (grading, washing, peeling, and dicing) were the same for both industrial lines. The tomatoes were peeled using a steam system (Cavalieri, Italy) under pressurized steam at 110 °C for 10 s, followed by mechanical separation of the peels. A linear dicer was used to cut tomatoes (Cavalieri, Italy). The tomatoes were first cut into 2 cm thick slices, and afterward to 1.7 cm-side cubes. Diced tomatoes and juice were mixed in a tank for approximately 10 min for further processing by appertization or ohmic heating. A volumetric pump was used to feed the ohmic heating and appertization units. This pump minimizes product damage and enables moving fluid–solid mixtures containing approximately 60% of diced tomatoes in 40% tomato juice. Figures 1 and 2 illustrate the system boundaries in CTwJ processing and packaging upon appertization and ohmic heating systems.

### Appertization line

Diced tomatoes and juice were mixed in a tank for 10 min. They were pre-heated (ca. 60 °C) using direct steam injection before hot filling in tinplate cans. Cans sterilization was done using a tunnel sterilizer (Manzini, Italy) at 95 °C for 30 min with a production rate of 6 t/h of canned diced tomatoes with juice. The steam production system consists of firetube steam boilers (PB 100 model, Mingazzini, Parma, Italy) with a steam output of 10,000 kg/h and rated pressure of 12 bar.

### Aseptic ohmic heating line

The process is based on the electrical resistance of the chopped tomato with the juice that is treated. Dissipation of the electrical energy when electric current flows through food leads to heat release (Joule effect)^40^. Ohmic heating was conducted in an industrial-scale unit, consisting of one stage for pre-heating the CTwJ up to 75 °C and a subsequent stage for heating them up to 102 °C. It then enters the insulated holding tubes before being cooled in tubular heat exchangers (Tetra Pak, Denmark) up to 20 °C. After cooling, the product is pumped into the aseptic storage vessel prior to aseptic packaging in multi-layer plastic pouches. The total electrical power of the ohmic system was 240 kW, and the average product flowrate was 4 t/h. The flowrate was recorded using an electromagnetic flowmeter (EMC, Auckland, New Zealand) with a precision of within 1% of the full range. The bulk temperatures were measured using platinum resistance probes (Pt 100 Ω to 0 °C with a ± 0.1 °C accuracy) placed at the inlet and outlet of each zone. Relative pressure was measured with manometers (JUMO, type 4AP30, Fulda, Germany) at the inlet and outlet of each zone with a precision of 0.1%. The power supply delivers bipolar potential pulses, and electrolysis is prevented by using a high-frequency alternating voltage^41^.

### Energy auditing

Energy requirements were experimentally measured through energy auditing. Both processing lines were instrumented to determine thermal and electrical energy inputs and consumption.

The energy requirement for the appertization line encompasses the electricity used by conveyers, pumping systems, and line dicers; thermal energy is required for peeling, pre-heating prior to appertization, sterilization and cooling of the product.

The specific energy required for heating the cans (*E*_*c*_) and their contents (*E*_*p*_) was calculated using Eqs. (1) and (2), where *n*_*c*_ is the number of cans per cycle, *m*_*c*_ is the weight of empty cans, *m*_*p*_ is the weight of the product per can, *C*_*pc*_ is the specific heat of cans, *C*_*pp*_ is the specific heat of the product, *T*_*c*_ is the temperature of the cans, *T*_*p*_ is the temperature of the product, and *T*_*s*_ is the steam temperature inside the sterilizer:

1 $$E_{c} = n_{c} \times m_{c} \times C_{{pc}} \times (T_{s} - T_{c} )$$

2 $$E_{p} = n_{c} \times m_{p} \times C_{{pp}} \times (T_{s} - T_{p} )$$

The energy input in the tunnel sterilizer (*E*_*s*_) was calculated using Eq. (3), with $\dot{m}_{{vs}}$ as the steam mass flowrate, and *h*_*vs*_ is the specific enthalpy of steam:

3 $$E_{s} = \dot{m}_{{vs}} h_{{vs}}$$

Ohmic heating occurs due to the electrical conductivity and ability of the electrical current to flow through it. The power clamp meter was connected to the electrical power supply to measure the given electrical power (*P*_*g*_). The fraction of electrical power converted to thermal energy and dissipated in the product was calculated using Eq. (4), with $\dot{m}_{{po}}$ as the mass flowrate of the product in the ohmic heater, *C*_*pp*_ is the specific heat of the product, *T*_*pi*_ is the inlet temperature of the product, and *T*_*po*_ is the outlet temperature of the product.

4 $$P_{t} = \dot{m}_{{po}} \times C_{{pp}} \times (T_{{po}} - T_{{pi}} )$$

The energy required for final cooling prior to aseptic packaging was calculated using Eq. (5), where $\dot{m}_{{wc}}$ is the mass flowrate of cooling water, *C*_*pw*_ is the specific heat of water, *T*_*iw*_ is the inlet temperature water, and *T*_*ow*_ is the outlet temperature of water in the heat exchanger:

5 $$P_{t} = \dot{m}_{{wc}} \times C_{{pw}} \times (T_{{ow}} - T_{{iw}} )$$

### Software

EDraw Max (ver. 9.1, 2018; Sheung Wan, Hong Kong) was used for the representation of the appertization and ohmic heating flowcharts. The LCA analysis was performed using the professional SimaPro software (ver. 8.1.0 Analyst) and adapted Ecoinvent 3.2 database.

### LCA methodology

LCA is a standardized and widespread methodology to study environmental consequences associated with food^42^. The LCA procedure is outlined by ISO 14040 and ISO 14044^43^. A comprehensive LCA comprises four coherent and iterative phases (1) goal and scope definition, (2) life cycle inventory, (3) impact assessment, and (4) interpretation of the results.

#### Definition of the goal and scope

The objective of this study was to perform an attributional life cycle environmental assessment of CTwJ processing and packaging upon appertization and ohmic heating systems. The functional unit (FU) was considered as 1 kg of CTwJ, which is a single reference of the product, requiring 1.6 kg of fresh tomato. Mass-based FU is common in food processing LCA (see Table 1). Figures 1 and 2 illustrate the system boundaries in CTwJ processing and packaging for the appertization and ohmic heating systems.

#### Life cycle inventory

The inputs and outputs of the investigated system were quantified in the second phase of the LCA^44^. The cradle-to-grave emissions were classified into background (off-site) and foreground (on-site) emissions^45^. The background emissions include the emitted pollutants from the production of material inputs, for instance, the emissions released within the generation of electricity and natural gas. However, foreground emissions comprise the direct emissions from the consumption of inputs in the investigated factory, for instance, emissions released within the combustion of natural gas. The background’s emissions coefficients, such as the emissions of electricity generation and distribution, were adapted from the Ecoinvent database.

#### Life cycle impact assessment

In the third phase of an LCA study, impact category selection and characterization are mandatory; however, normalization and weighing are optional^46^. The IMPACT 2002+ methodology was applied as the baseline impact assessment methodology, given its inclusion of various impact and damage categories. It is also a combination hybrid IMPACT 2002, Eco-Indicator 99, CML, and IPCC.

### Uncertainty analysis of the impact assessment

There are some sources of uncertainties affecting the LCA results, including data quality, scenarios, and mathematical models underlying the impact assessment methods^44,47^. As shown in Table 1, studies on the LCA of tomato processing have employed different impact assessment methodologies (see Table 1). In this study, an uncertainty analysis was performed to investigate the effect of impact assessment selection on the LCA results of the case study. For this purpose, six impact assessment methodologies, i.e., EDIP 2003, CML-IA baseline, EDP 2013, ILCD 2011 Midpoint, ReCiPe midpoint, and IMPACT 2002+ were considered to be evaluated by LCA. The analyses were conducted using SimaPro V8.0.3.14.

### Research involving plants

Studies complied with local and national regulations for using plants.

## Results and discussion

### Energy auditing

An overview of the energy and water requirements for the aseptic ohmic heating and appertization lines is presented in Table 2.

**Table 2:** Overview of energy and water consumption for producing 1 kg of CTwJ through industrial appertization and aseptic ohmic heating lines.

	Appertization line	Aseptic Ohmic heating line
**Pre-processing**		
Electricity, Wh/kg	0.23	0.19
Natural gas, m^3^/kg	7.92 × 10^–4^	2.38 × 10^–5^
Water, m^3^/kg	0.37	0.34
**Processing**		
Electricity, Wh/kg	3.19	53.97
Natural gas, m^3^/kg	1.48 × 10^–2^	–
Water, m^3^/kg	1.32	1.35

The global specific energies for appertization and ohmic heating industrial lines for pre-processing and processing of CTwJ were 156 and 54 kWh/t, respectively. The energy requirement for the appertization line encompasses the electricity consumed by conveyers, pumping systems, and line dicers; the thermal energy required for peeling, pre-heating prior to appertization, and the energy required for sterilization, cooling, and packing of the product. Energy uptake for the ohmic heating includes the electricity used by conveyers, pumping systems, and line dicers; thermal energy required for peeling, ohmic pre-heating and ohmic sterilization; and energy consumption for cooling and aseptic packaging of the final product. The energy saving of the ohmic heating line compared to the appertization line is 102 kWh/t of the product (or 65% energy saving).

The energy efficiencies of the appertization and ohmic heating systems are 25% and 77%, respectively. For ohmic heating, the electrical energy input is converted to thermal energy by the Joule effect, where the chopped tomato with juice behaves like a resistor in an electrical circuit. Energy losses in the ohmic heating system are mainly due to the pre-processing step and the cooling of the product during the holding phase between ohmic pre-heating and ohmic heating steps of the product; this temperature gradient was around 4.5 °C. Adding thermal insulation to this holding zone will improve the energy efficiency of the ohmic heating system.

Energy losses in the appertization line are mainly due to the lack of insulation, lack of reuse of steam condensate, and non-condensation of part of the injected steam. Steam condensate could be reused to heat feedwater for the steam boiler, pre-heat utilities, or clean equipment. Another major reason is the non-synchronization of the cans flow rates between the filling and retorting levels, which induces a continuous injection of steam in the retort even with a low load of cans. This situation leads to large energy losses due to the high steam consumption. This low efficiency can be improved by installing a steam regulation in the retort, which will provide the required steam flow depending on the load of the cans.

### Interpretation of LCA results

Table 3 displays the characterization indices for CTwJ production. The global warming potential, ozone layer depletion, and non-renewable energy consumption for 1 kg production of packaged CTwJ for the appertization system were determined to be 4.38 kg CO_2_eq, 1.34 × 10^–7^ kg CFC-11 eq, and 52.15 MJ, respectively. However, the aforementioned amounts for 1 kg of packaged CTwJ production upon the ohmic heating system were 2.52 kg CO_2_eq, 4.00 × 10^–8^ kg CFC-11 eq, and 24.94 MJ, respectively. The results clearly showed that the environmental impacts of CTwJ processing and packaging on appertization were higher than those of ohmic heating systems. In other words, CTwJ production by the ohmic heating system was more environmentally efficient. The global warming potential of tomato sauce production was reported to be 1.5 kg CO_2_eq^48^.

**Table 3:** Characterization indices for tomato juice production.

Impact category	Unit	Appertization	Ohmic heating
Global warming	kg CO_2_ eq	4.38	2.52
Mineral extraction	MJ surplus	67.11	0.02
Non-renewable energy	MJ primary	52.15	24.94
Ozone layer depletion	kg CFC-11 eq	1.34 × 10^–7^	4.00 × 10^–8^
Ionizing radiation	Bq C-14 eq	55.49	10.96
Respiratory organics	kg C_2_H_4_ eq	0.0019	0.001
Respiratory inorganics	kg PM_2.5_ eq	0.01	0.002
Non-carcinogens	kg C_2_H_3_Cl eq	0.11	0.03
Carcinogens	kg C_2_H_3_Cl eq	0.08	0.03
Aquatic ecotoxicity	kg TEG water	1943.03	505.51
Terrestrial acid/nutri	kg SO_2_ eq	0.15	0.029
Terrestrial ecotoxicity	kg TEG soil	526.11	161.90
Aquatic acidification	kg SO_2_ eq	0.06	0.01
Aquatic eutrophication	kg PO_4_ P-lim	0.0006	0.0003
Land occupation	m^2^org.arable	0.20	0.09

**Figure 3:**
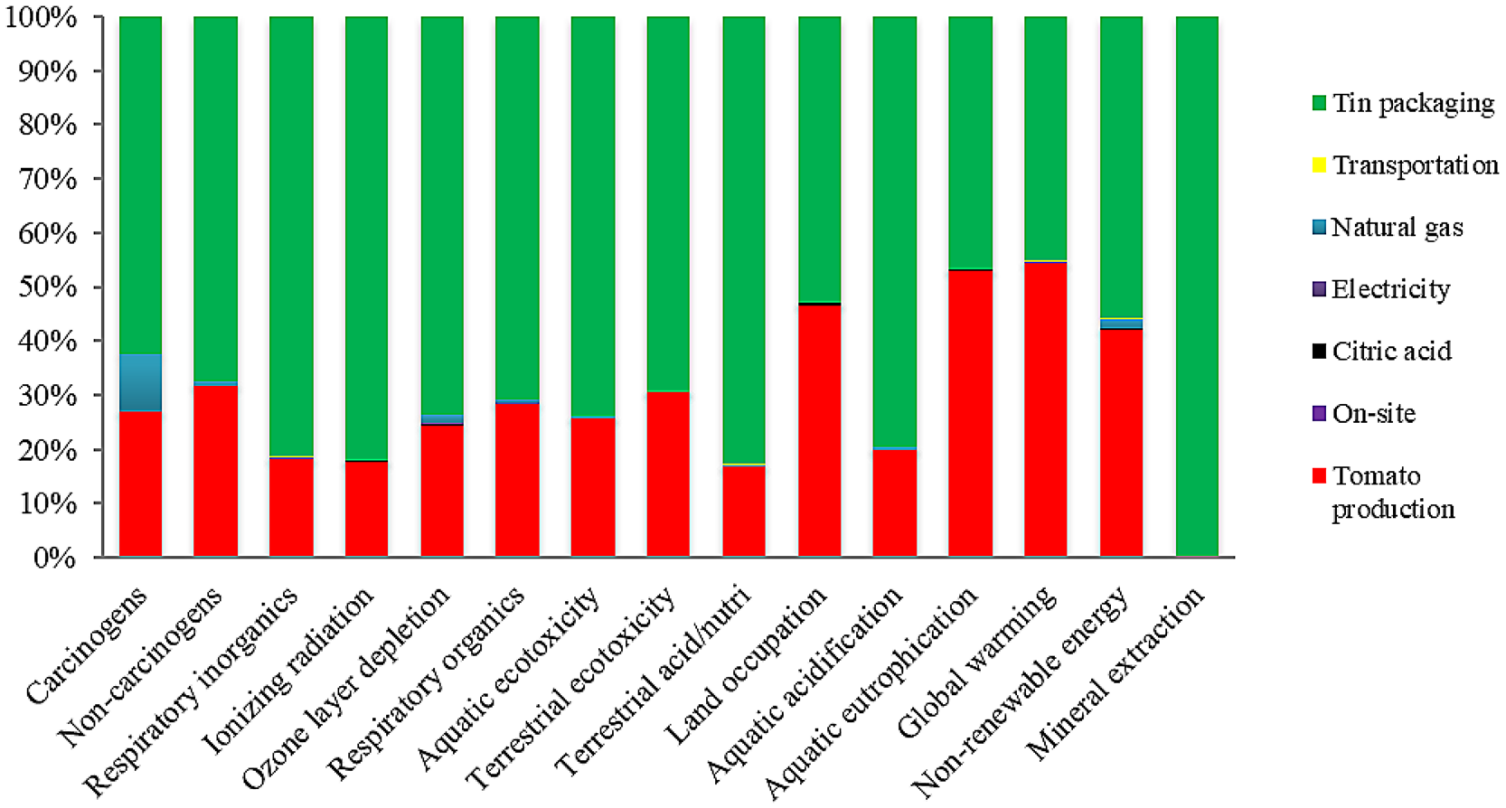
Inputs contribution to the environmental impact of tomato juice production upon appertization.

**Figure 4:**
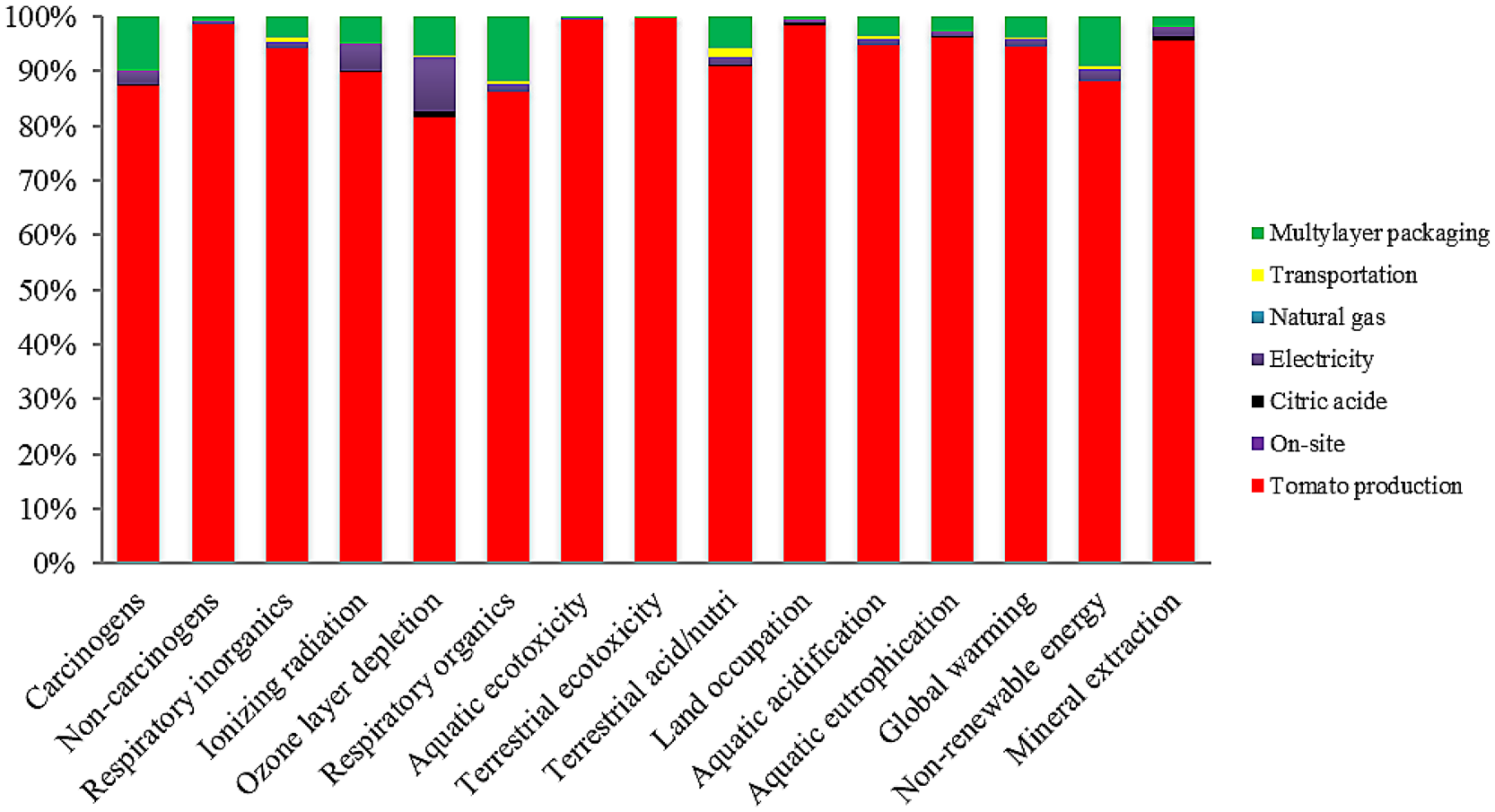
Inputs contribution to the environmental impact of tomato juice production upon ohmic heating.

Figures 3 and 4 illustrate the share of inputs in environmental burdens of CTwJ production upon appertization and ohmic heating systems, respectively. The results indicated that the tin production phase was the hotspot in CTwJ production by the appertization system. The agricultural phase was the second main contributor to the most impacted categories. More specifically, the agricultural phase of tomato production and tin packaging accounted for 54.33% and 45.00% of the total global warming potential of CTwJ production, respectively. The results are in line with the study of Manfredi and Vignali^49^, which indicated that packaging was the main contributor to most impact categories for tomato purée production in a glass jar. Arnal et al.^50^ also highlighted that canning was the largest contributor to the total industrial-scale environmental impacts of packaged peeled tomato production systems. Packaging was reported to be responsible for the environmental impacts within all selected impact categories of tomato purée production, except for acidification and eutrophication^51^. Del Borghi et al.^13^ showed that packaging and agricultural phases had the highest adverse impacts on the environment during the production of 13 different tomato processed products. Many studies have also reported that the packaging phase is an environmental hotspot during the production of some other processed food products, such as dairy products^52^, and canned sardine^53^.

The results highlight the important contributions of the agricultural phase in the ohmic heating system in the most impacted categories. The agricultural phase accounted for 94.44% of the total global warming potential of CTwJ production by the ohmic heating system. In other words, the consumption of inputs, such as diesel fuel and chemical fertilizer cause a huge negative environmental impact during the CTwJ production supply chain.

**Figure 5:**
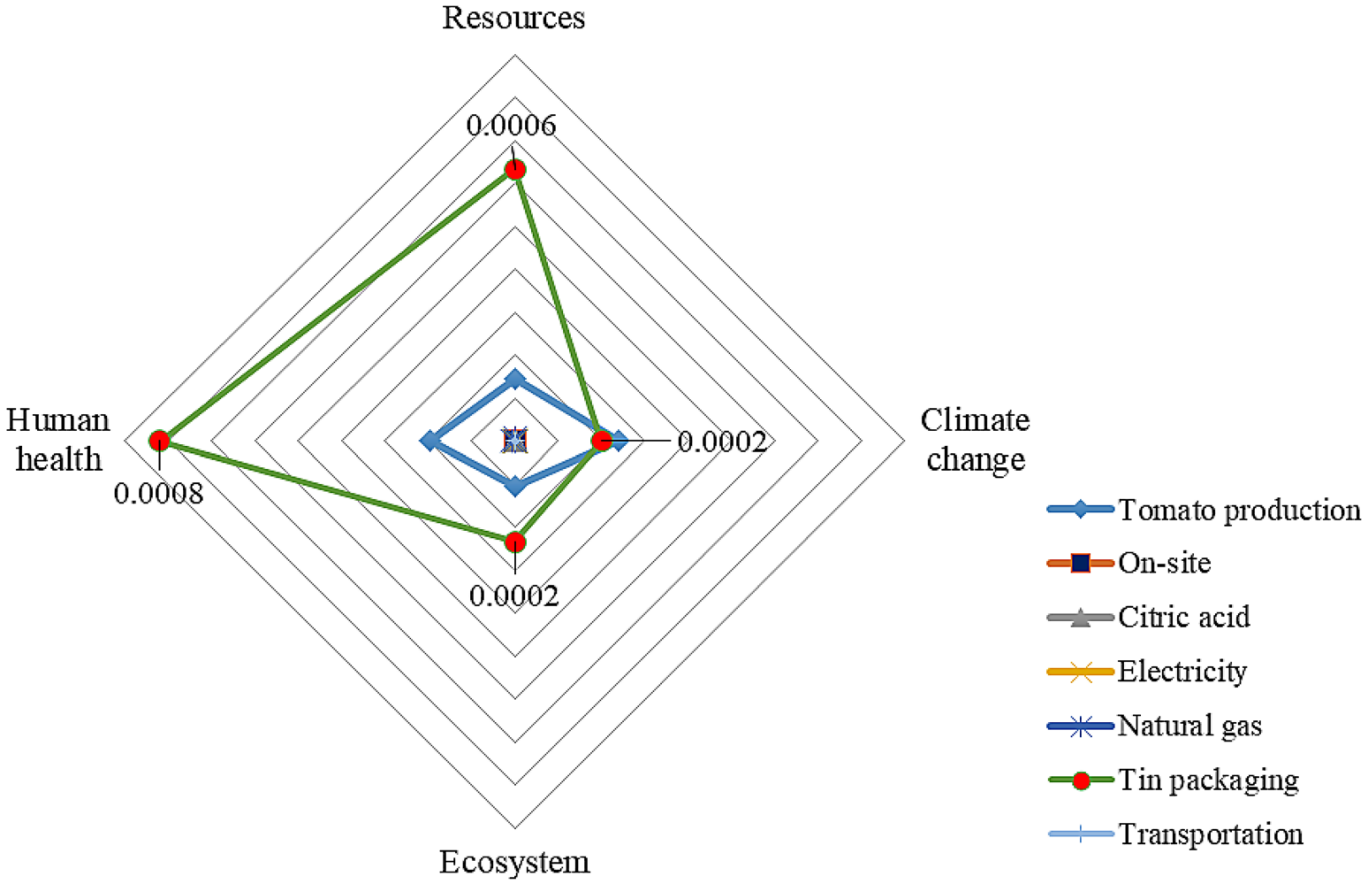
Normalized values of input damages in CTwJ processing and packaging for the appertization system.

**Figure 6:**
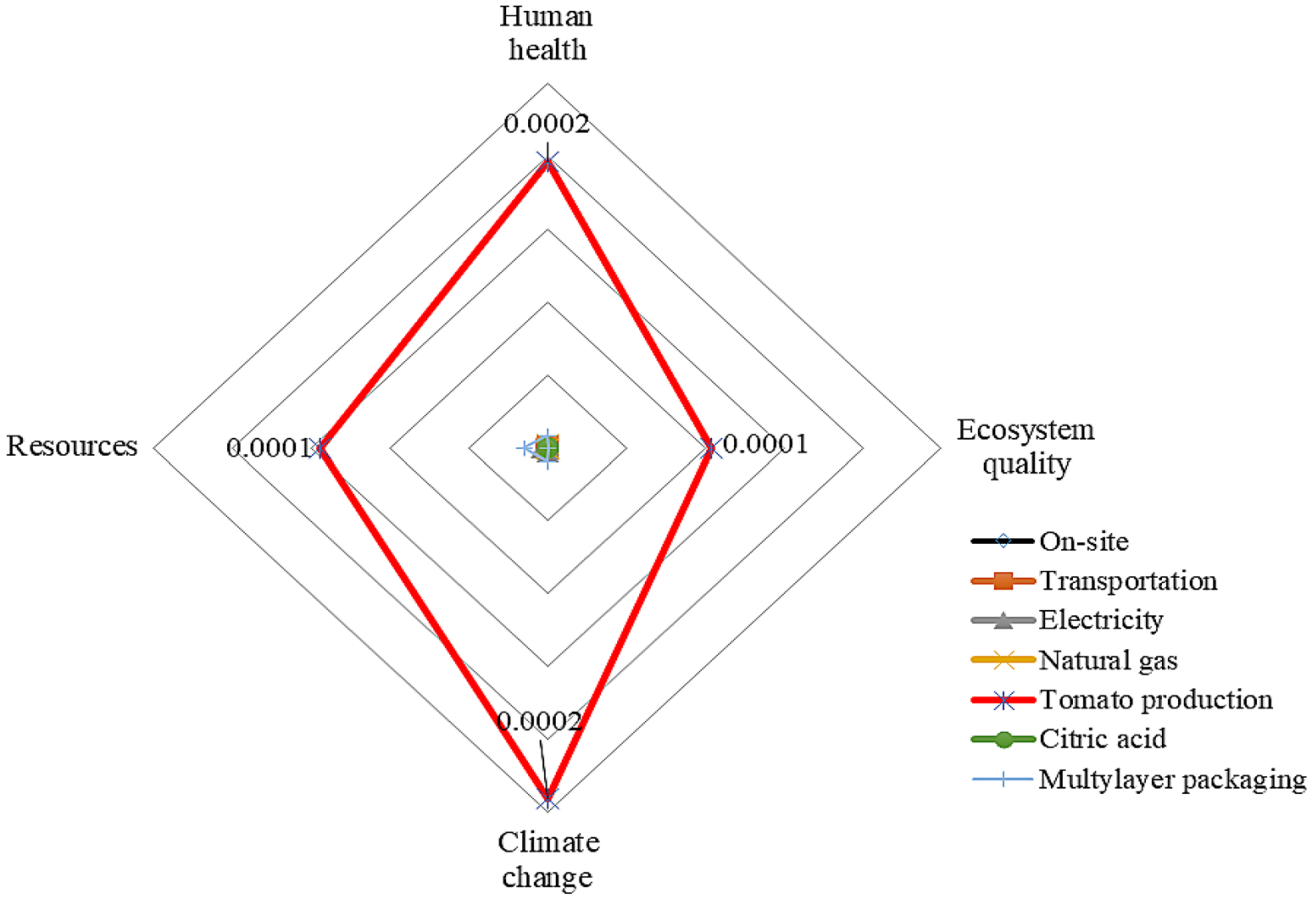
Normalized values of inputs damages in CTwJ processing and packaging for the ohmic heating system.

Figure 5 and 6 show the normalized damage assessment of CTwJ processing and packaging upon appertization and ohmic heating systems, respectively. The largest adverse environmental impact belonged to the human health damage category upon CTwJ production in both systems. The direct emissions of fossil fuels during the tomato production supply chain play a key role in the human health damage category. The resources damage category was placed as the second damage category with higher adverse environmental impacts in packed CTwJ production.

**Figure 7:**
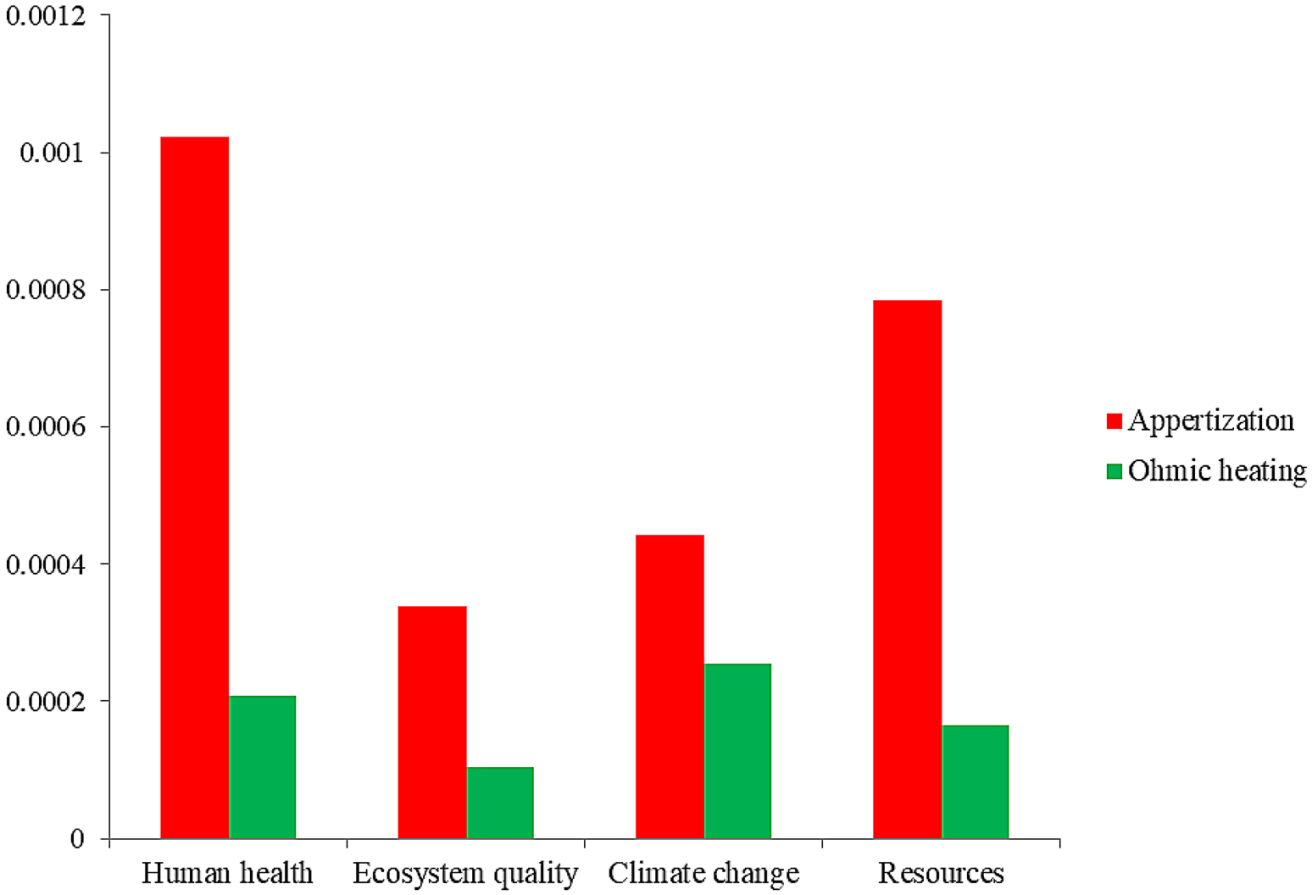
Normalized damage assessment of tomato juice processing and packaging for appertization and ohmic heating systems.

Figure 7 illustrates the normalized damage assessment of CTwJ processing and packaging for appertization and ohmic heating systems. The normalized damage assessment of CTwJ processing and packaging for the appertization system was higher than the ohmic heating system in all impact categories. So, the packed CTwJ for the ohmic heating system was more environmentally friendly. An LCA study on tomato processing also indicated that the application of PEF technology could mitigate environmental impacts^50^.

### Uncertainty analysis of the impact assessment

Table 4 illustrates the indicators of CTwJ production for different impact assessment methodologies. The obtained results help to compare the results of this study with those published on LCA tomato-based products. The results showed that the global warming potential of 1 kg of packaged CTwJ ranges from 4.13 to 4.44 kg CO_2_eq, within the appertization line. In addition, the global warming potential of the ohmic heating system ranges from 2.50 to 2.54 kg CO_2_eq. The results also indicated that the ozone layer depletion for the production of 1 kg of packaged CTwJ ranges from 1.33 × 10^–7^ to 1.35 × 10^–7^ kg CFC-11 eq; however, this value for ohmic heating systems ranges from 3.99 × 10^–8^ to 4.03 × 10^–8^.

**Table 4:** Total characterized indicators of CTwJ within the selected impact assessment methodologies.

Impact assessment	Global warming (kg CO_2_eq)		Ozone layer depletion (kg CFC-11 eq)	
	Appertization	Ohmic heating	Appertization	Ohmic heating
CML-IA baseline	4.44	2.50	1.34 × 10^–7^	4.00 × 10^–8^
EDIP 2003	4.44	2.50	1.34 × 10^–7^	4.00 × 10^–8^
EDP (2013)	4.44	2.50	1.34 × 10^–7^	4.00 × 10^–8^
ILCD 2011 Midpoint	4.44	2.50	1.33 × 10^–7^	3.99 × 10^–8^
ReCiPe midpoint	4.13	2.54	1.35 × 10^–7^	4.03 × 10^–8^
IMPACT 2002+	4.38	2.52	1.34 × 10^–7^	4.00 × 10^–8^

### Mitigation strategies

As previously mentioned, packaging and agricultural phases were the environmental hotspots in both systems. One solution to reduce the environmental burdens of tomato product processes is related to the packaging phase; it could be weight reduction^49^, and transitioning to packaging materials with less environmental impacts, such as bio-based packaging^13,51^. Therefore, the mitigation of the environmental impacts of tomato cultivation, replacing the packaging materials with bio-based materials, and using the by-products of cultivation and processing for supplying a part of the energy requirement can be considered as the main strategy for environmental impact mitigation of the CTwJ production supply chain. In the case of agricultural phase of CTwJ supply chain, Muñoz et al.^28^ highlighted that the environmental impacts of 1 kg of tomato production in a greenhouse is less than that of an open field system due to the efficient use of water, fertilizers, and pesticides. A study compared three tomato farming systems (open field, greenhouse, and hydroponic) in terms of energy use patterns and concluded that the hydroponic system was the most environmentally friendly system^54^. Bojacá et al.^24^ believed that the implementation of integrated pest management programs could mitigate the environmental impacts of Colombian greenhouse tomato production. Bacenetti et al.^55^ compared the two scenarios of the tomato purée production supply chain. In the first scenario, tomato by-products were sent back to the farms as bio-fertilizers; in the second scenario, the by-products were used in terms of biogas generation. The results showed that the second scenario was more environmentally efficient.

Moreover, there are some measures which can be taken in to consideration in order to mitigate the environmental impacts of CTwJ processing. For instance, a study focusing on the valorization of tomato by-products (tomato seeds and peels) highlighted the potential application of whole tomato by-products for valuable compound recovery and sequential low-cost biosorbent production^56^. Winans et al.^57^ applied LCA to study the diced tomatoes and paste production systems in California over a 10-year timeframe. They showed that the introduction of renewables in the life cycle of the production systems, such as solar-powered irrigation pumps, and on-site solar energy generation for facilities can mitigate the GWP impacts by 9–10%.

## Conclusions

This is the first study to compare the energy requirement and life cycle environmental impact of a novel food processing technology (ohmic heating) with a conventional method (appertization) in the tomato processing industry, considered one of the largest food processing industries worldwide. Moreover, uncertainty analysis was performed through the application of six different impact assessment methodologies.

The energy requirement evaluation showed the highest energy consumption for appertization (156 kWh/t of product). The energy saving of the ohmic heating line compared to the appertization line was 102 kWh/t of the product (or 65% energy saving). The energy efficiencies of the appertization and ohmic heating systems are 25% and 77%, respectively. There are opportunities for energy optimization of the investigated processes while maintaining the potential quality benefit. In the appertization system, a more energy-efficient process could be obtained by reducing steam non-condensation and installing a steam regulation, which will give the necessary steam flow depending on the load of the cans. In the case of ohmic heating, adding thermal insulation to the holding zone will improve the energy efficiency of the system.

From the LCA perspective, the uncertainty analysis results suggested that the global warming potential of the production of 1 kg of packaged CTwJ ranges from 4.13 to 4.44 kg CO_2_eq. In addition, the global warming potential of the ohmic heating system ranges from 2.54 to 2.78 kg CO_2_eq. Overall, CTwJ production by the ohmic heating system was more environmentally efficient than traditional retort canning.

Given this study was conducted on an industrial scale, the effect of influencing variables in the manufacture process of CTwJ production was not optimized. Therefore, further research is needed to optimize the processes in terms of energy and environmental impacts. In addition, replacing the packaging materials with bio-based materials, and using the by-products of cultivation and processing for supplying a part of the energy requirement, on the final energy and environmental impacts of CTwJ production should be further explored.

## Abbreviations

CML: Institute of environmental sciences
CO_2_: Carbon dioxide
CTwJ: Chopped tomatoes with juice
Eq: Equivalent
FU: Functional unit
FU: Functional units
HPP: High pressure processing
IPCC: Intergovernmental panel on climate change
ISO: International standardization organization
kg: Kilogram
LCA: Life cycle assessment
LCI: Life cycle inventory
m^3^: Cubic meter
MJ: Mega joule
PEF: Pulsed electric fields
SO_2_: Sulfur dioxide
t: Tonne
Wh: Watt-hour
